# Avocado consumption during pregnancy linked to lower child food allergy risk: prospective KuBiCo study

**DOI:** 10.1038/s41390-025-03968-4

**Published:** 2025-03-07

**Authors:** Feon W. Cheng, Ella Bauer, Nikki A. Ford, Katri Backman, Raimo Voutilainen, Markku Pasanen, Leea Keski-Nisula, Sari Hantunen

**Affiliations:** 1 Avocado Nutrition Center, Hass Avocado Board, 25212 Marguerite Pkwy #250, Mission Viejo, CA 92692 USA; 2 Institute of Clinical Medicine, School of Medicine, University of Eastern Finland, FI-70211 Kuopio, Finland; 3 Department of Pediatrics, Kuopio University Hospital, FI-70211 Kuopio, Finland; 4 School of Pharmacy, University of Eastern Finland, FI-70211 Kuopio, Finland; 5 Department of Obstetrics and Gynecology, Kuopio University Hospital, FI-70211 Kuopio, Finland; 6 Institute of Public Health and Clinical Nutrition, University of Eastern Finland, P.O. Box 1627, FI-70211 Kuopio, Finland

## Abstract

**Background:**

Maternal exposures during pregnancy play a critical role in offspring’s health outcomes. This study aimed to investigate how maternal avocado consumption during pregnancy relates to offspring allergic health outcomes using the Kuopio Birth Cohort (KuBiCo) Study.

**Methods:**

This prospective cohort study used data from KuBiCo. Avocado consumption was assessed using an online food frequency questionnaire in trimesters (T) 1 and 3. Avocado consumers were defined as participants who reported consuming any avocado (>0 grams) in T1 and/or 3, and avocado non-consumers were defined as those who didn’t report consuming any avocado (0 grams) in both T1 and 3. The 12-month follow-up questionnaire captured offspring allergic outcomes (rhinitis, paroxysmal wheezing, atopic eczema, and food allergy).

**Results:**

Of 4647 participants, 2272 met the criteria and were included in the analysis. Compared to avocado non-consumers (during pregnancy), avocado consumers (during pregnancy) had 43.6% lower odds of reporting food allergy among their children at the 12-month follow-up questionnaire while adjusted for relevant covariates. No significant associations were noted in the other three allergic health outcomes in the fully adjusted model.

**Conclusion:**

Avocado consumption during pregnancy was associated with lower odds of infant food allergies at 12 months, even when accounting for potential covariates.

**Impact:**

- Maternal exposures, such as nutrition during pregnancy, can affect offspring health outcomes. Consuming certain nutrients, which are found in avocados, during pregnancy have been associated with lower allergic health outcomes in children.
- Avocado consumption during pregnancy is found to be associated with lower odds of infant food allergies at 12 months, even when accounting for potential covariates.

## Introduction

Maternal exposures, such as nutrition and lifestyle during pregnancy, play a critical role in an offspring’s health outcomes. This includes immunoglobulin E-mediated allergic diseases (or atopic diseases), which encompasses conditions like allergic rhinitis, eczema (or atopic dermatitis), asthma, and wheezing.^1,2^ Research has noted that different diets or foods during pregnancy may have varying impacts on offspring’s allergic health outcomes. A maternal confectionary diet made up largely of baked and sugary foods may lead to an increased food allergy development in the infant.^3^ Consuming nutrient poor, proinflammatory diet during pregnancy was also linked to an increased risk of asthma development in their children.^4^ On the other hand, Venter et al. found that 4-year-old children had fewer allergic outcomes, including allergic rhinitis, eczema, asthma, and wheezing, when their mothers adhered to a higher maternal diet index characterized by higher intakes of yogurt and vegetables during pregnancy.^5^ Similarly, Chatzi et al. found that a high Mediterranean Diet Score during pregnancy was protective against the development of allergic wheezing and persistent wheezing in offspring at 6.5 years old.^6^ Although the exact mechanism is still being examined, the literature suggests that antioxidant compounds from foods, such as fruits and vegetables, provide immunomodulating benefits during this critical time for immune development.^7^

Maternal consumption of individual foods has also been associated with allergic outcomes in offspring. For instance, higher consumption of processed meat or meat products during pregnancy was linked to a higher risk of wheezing within the first year of an infant’s life.^6^ Alternatively, Willers et al. found maternal apple intake during pregnancy was associated with lower wheezing and asthma among 5-year-old offspring. However, these authors found no significant associations between maternal total fruit intake during pregnancy and allergic respiratory outcomes in offspring.^8^ This may be due to the unique composition that each fruit has to offer. These findings are helpful when practitioners are trying to communicate practical applications to pregnant women, such as women are inquiring about which foods can help reduce their children’s risk of developing allergic diseases.

Avocados contain numerous vitamins, minerals, and phytochemicals, which are known to support immune and metabolic health.^9–11^ However, no study has examined the effects of avocado intake during pregnancy and allergic outcomes among children. Thus, this study aims to investigate how maternal avocado consumption during pregnancy relates to allergic health outcomes in offspring using the Kuopio Birth Cohort (KuBiCo) Study.

## Methods

### Study population

This prospective cohort study used data from KuBiCo (https://uefconnect.uef.fi/en/group/kuopio-birth-cohort-kubico/), initiated in 2012, with the objective of incorporating approximately 10,000 mother-child pairs. KuBiCo aims to enhance our understanding of the impact of genetic, lifestyle factors, and environmental conditions during pregnancy on maternal and offspring health outcomes.^12^ KuBiCo extended invitations to pregnant women anticipated to deliver at Kuopio University Hospital (KUH), Finland to join the study, and over 90% of participants were recruited during their routine first-trimester appointments.^12^ The collection of nutritional data began in March 2013. This study includes pregnant women who filled out nutrition questionnaires between March 2013 and November 2022.

The overall participation in KuBiCo during 2013–2022 (7944 women) has been 37.7% of all parturients giving birth in KUH. These participants reflected the overall population of women who gave birth, considering factors such as maternal age, gestational length, infant sex, and birth weight.^12^ Data collection included questionnaires, biological samples, and health examinations conducted at various points during pregnancy and at delivery. Following childbirth, KuBiCo maintains annual follow-ups involving questionnaires and health examinations for both the mother and child.^12^ The KuBiCo database is linked to the hospital birth register and later also national health registries for epidemiological follow-ups.^12^ Additional details of the KuBiCo Study have been published elsewhere.^12^

### Dietary assessment

Avocado consumption data were collected through an adapted online food frequency questionnaire (FFQ) administered during the first and third trimesters. The KuBiCo FFQ was adapted to include more current food items, comprising a total of 163 items, each with a predefined portion size (in grams). Respondents were asked how often (with nine frequency options) in the past three months they had consumed a food item (e.g., avocado). Portion size and frequency were used to compute an estimated daily intake for each listed item. Lastly, the 2018 National Food Composition (Fineli) database was utilized to determine average daily intakes of energy, food, and nutrients.^13^ This is a modified version of the FFQ used in the Kuopio Breast Cancer Study; its validity and reliability have been detailed by Männistö et al.^14^

KuBiCo participants were categorized as avocado consumers or non-consumers. Avocado consumers were defined as participants who reported consuming any avocado (>0 grams) during the first or third trimester, and avocado non-consumers were defined as participants who didn’t report consuming any avocado (0 grams) at both trimesters.

### Offspring allergic outcomes

Offspring allergic outcomes were captured in the 12-month follow-up questionnaire. KuBiCo participants were asked (yes/no): “Has your child experienced the following condition during his/her first year of life?” for rhinitis (other than during a cold), paroxysmal wheezing (i.e., acute episodes of wheezing). Self-reported physician-diagnosed conditions were also asked (yes/no): “Has a doctor diagnosed any of the following conditions in your child during his/her first year of life?” for eczema and food allergy.

The four allergic outcomes (rhinitis, paroxysmal wheezing, eczema, and food allergy) were categorized as binary variables (yes or no).

### Covariates

The following covariates were considered: maternal age at delivery (continuous), marital status (married, cohabitation, other relationship, divorced or widow, or single), educational level (16 years or less or more than 16 years), parity (nulli- or primiparous or multiparous), body mass index (BMI) at the first trimester (continuous), gestational age at delivery (continuous), caesarean section (yes or no), neonatal intensive care unit (yes or no), breastfeeding – number of months (continuous), alcohol consumption – percentage of total energy/day at trimesters 1 and 3 (continuous), Edinburgh Postnatal Depression Scale (EPDS) at postpartum (continuous), diet quality at trimesters 1 and 3 (continuous), and smoking (not smoking, stopping smoking at 1st trimester, smoking, not known, smoking before pregnancy, stopped smoking after 1st trimester, or passive smoking).

KuBiCo participants completed the EPDS through an online questionnaire at eight weeks postpartum. The EPDS, utilized for screening postpartum depression, is a 10-item questionnaire employing a 4-level Likert scale with scores ranging from 0 to 30.^15^

Diet quality was estimating using the Alternative Healthy Eating Index for Pregnancy (AHEI-P)^16^ based on the FFQ data in trimesters 1 and 3. The original AHEI is a valid measure of diet quality that focuses on food items associated with decreased chronic disease risk. To make AHEI more suitable for pregnant women, Rifas-Shiman et al. included folate, calcium, and iron components and excluded the alcohol component, and because some participants may avoid nuts due to allergic sensitization, the nuts and soy protein-component were excluded and tofu and soybeans were included in the vegetable component.^17^ The AHEI-P contains nine dietary components: vegetables, fruit, the ratio of white to red meat, fiber, trans fat, the ratio of polyunsaturated to saturated fatty acids, folate, calcium, and iron. Dietary recommendations for pregnant women determine the minimum and maximum points of each component, with a maximum score of 10 points for each component. The scores for the individual dietary components were summed to get the total AHEI-P score for each participant. The total AHEI-P score is on a 90-point scale, and a higher total AHEI-P score indicates better dietary quality.

### Statistical analyses

We performed descriptive statistics to compare characteristics between mothers who consumed avocados during pregnancy and those who did not. Maternal characteristics between avocado consumers and non-consumers were compared using chi-square tests and independent sample t-tests. We used logistic regression to examine the association between the two avocado consumer groups during pregnancy and the offspring’s allergic outcome. Model 1 was unadjusted. Model 2 was adjusted for maternal age at delivery, marital status, education, nulliparity, BMI in the first trimester, gestational age at delivery, caesarean section, neonatal intensive care unit admission, breastfeeding, alcohol consumption, EPDS scores, diet quality (AHEI-P), and smoking. Missing values were imputed with the mean adjusted method. All analyses were performed with IBM SPSS Statistics 29, Armonk, NY, and the level of significance was considered at *p* < 0.05.

### Protocol registration and checklist

We developed and registered our study protocol at: https://osf.io/7mrgx. Furthermore, this study also adhered to the Strengthening the Reporting of Observational Studies in Epidemiology (STROBE) checklist to ensure reporting quality.

## Results

**Fig. 1:**
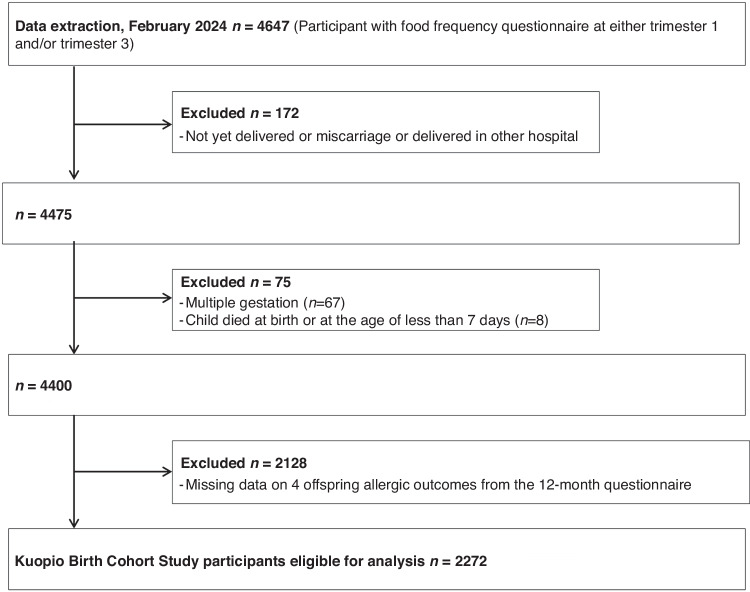
Flow chart of eligible Kuopio Birth Cohort (KuBiCo) Study participants included in this analysis. The four allergic outcomes include rhinitis, paroxysmal wheezing, eczema, and food allergy.

Of the 4647 participants, 2272 met the criteria and were included in the final analysis (Fig. 1). Table 1 describes the maternal characteristics from the KuBiCo cohort. On average, the maternal age at delivery was 31.3 years, with more than half of the participants being married. 56.9% of the mothers had completed more than 16 years of education, and over 80% were non-smokers. Most were nulliparous or primiparous, and the average BMI in the first trimester was approximately 25.1 kg/m^2^. The mean (standard deviation) scores for EPDS and diet quality were 4.5 (4.0) and 58.8 (10.2) in the whole study population, respectively.

**Table 1:** Maternal characteristics of participants from the Kuopio Birth Cohort Study, Finland by avocado consumer vs non-consumer (*n* = 2272)^a^.

Maternal characteristics^b^	All (*n* = 2272)	Avocado non-consumer during pregnancy (*n* = 1238)	Avocado consumer during pregnancy (*n* = 1034)	*P*-value
Maternal age at delivery (year)	31.1 (4.7)	30.8 (5.0)	31.5 (4.3)	0.001
Marital Status, %				0.062
Married	56.3	54.6	58.3	
Cohabitation	37.5	38.4	36.5	
Other relationship	3.0	3.8	2.0	
Divorced or widow	3.1	3.1	3.1	
Single	0.1	0.2	0.1	
Education, %				0.007
16 years or less	43.1	45.6	40.0	
More than 16 years	56.9	54.4	60.0	
Parity, %				<0.001
Nulli- or primiparous	83.4	80.3	87.1	
Multiparous	16.6	19.7	12.9	
Body mass index at the first trimester (kg/m^2^)	25.1 (5.0)	25.6 (5.3)	24.4 (4.5)	<0.001
Gestational age at delivery (weeks)	39.4 (1.5)	39.3 (1.5)	39.4 (1.5)	0.304
Caesarean delivery, %	13.6	15.4	11.6	0.013
Admitted to neonatal intensive care unit, %	11.5	11.9	11.1	0.501
Breastfeeding (months)	9.7 (3.5)	9.2 (3.8)	10.3 (3.0)	<0.001
Alcohol consumption (percentage of total energy/day)	0.008 (0.1)	0.01 (0.1)	0.006 (0.06)	0.303
EPDS scores	4.5 (4.0)	4.6 (4.1)	4.4 (3.8)	0.321
Diet quality (AHEI-P)	58.8 (10.2)	55.9 (10.3)	62.2 (8.8)	<0.001
Smoking during pregnancy, %				<0.001
Not smoking	81.1	77.1	86.0	
Stopping smoking at 1^st^ trimester	9.5	11.2	7.4	
Smoking	1.5	2.3	0.6	
Not known	0.7	1.0	0.5	
Smoking before pregnancy	3.7	4.4	2.7	
Stopped smoking after 1^st^ trimester	0.6	0.8	0.3	
Passive smoking	2.9	3.2	2.5	

*AHEI-P* Alternative Healthy Eating Index for Pregnancy, *BMI* body mass index, *EPDS* Edinburgh Postnatal Depression Scale.

^**a**^Means (standard deviation) for continuous variables and percentages for categorical variables.

^**b**^Avocado consumers were identified as Kuopio Birth Cohort Study participants who reported consuming any amount of avocado in their food frequency questionnaire at trimester 1 and/or trimester 3.

Mothers who included avocados in their pregnancy diet, in comparison to those who did not, tended to be older at delivery and less likely to undergo a caesarean delivery. They were also more likely to be non-smokers and have nulli- or primiparous pregnancy. Furthermore, avocado consumers exhibited higher scores for dietary quality, breastfed for a longer duration, and had lower BMI levels.

**Fig. 2:**
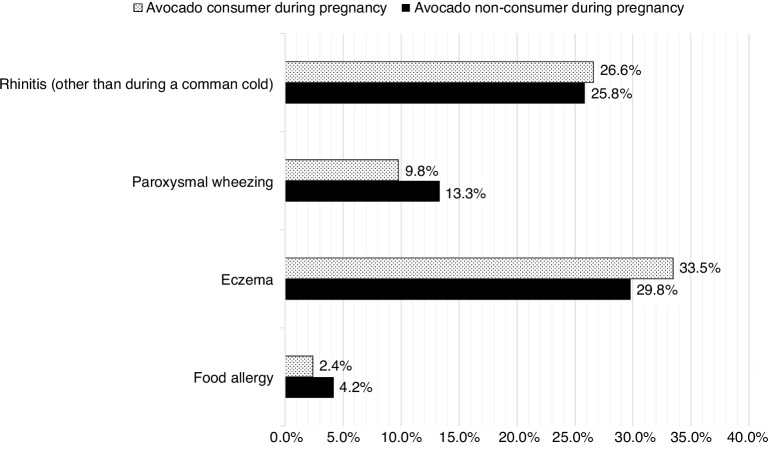
Percentage of reported offspring allergic diseases by avocado consumer vs. non-consumer from the eligible Kuopio Birth Cohort (KuBiCo) Study participants. The bar fill patterns distinguish between avocado consumers and non-consumers during pregnancy. A dotted pattern represents consumers, while a solid fill indicates non-consumers.

Compared to avocado non-consumers (during pregnancy), avocado consumers (during pregnancy) had 43.6% lower odds of having food allergy among their infants at the 12-month follow-up questionnaire while adjusted for relevant covariates, which included maternal age at delivery, marital status, education, nulliparity, BMI in the first trimester, gestational age at delivery, caesarean section, neonatal intensive care unit admission, breastfeeding, alcohol consumption, postpartum depression, diet quality, and smoking (Table 2). Food allergy was significantly higher (*p* = 0.019 in the fully adjusted model) in the offspring of pregnant non-consumers (4.2%) compared to the offspring of pregnant avocado consumers (2.4%) (Table 2 and Fig. 2). Similarly, compared to the offspring of gestational avocado consumers (9.8%), offspring of gestational non-consumers (13.3%) had significantly higher paroxysmal wheezing (*p* = 0.009 in unadjusted model). However, this association attenuated in the fully adjusted model (*p* = 0.085). No significant associations were noted in the other two allergic health outcomes (rhinitis and eczema).

**Table 2:** Odds Ratio (OR) and 95% confidence intervals (CI) of reported offspring allergic diseases comparing between avocado consumer and non-consumer during pregnancy^a^.

	Model 1^b^	Model 2^c^
**Rhinitis (other than during a common cold)**		
*N*	2272	2258
Odds Ratio	1.039	1.137
95% CI	0.862–1.254	0.928–1.395
*P*-value	0.687	0.216
**Paroxysmal wheezing**		
N	2272	2270
Odds Ratio	0.704	0.779
95% CI	0.541–0.915	0.587–1.035
*P*-value	0.009	0.085
**Eczema**		
N	2272	2270
Odds Ratio	1.184	1.143
95% CI	0.992–1.414	0.945–1.382
*P*-value	0.062	0.168
**Food allergy**		
N	2272	2272
Odds Ratio	0.565	0.564
95% CI	0.348–0.917	0.336-0.946
*P*-value	0.021	0.030

*AHEI-P* Alternative Healthy Eating Index for Pregnancy, *BMI* body mass index, *EPDS* Edinburgh Postnatal Depression Scale.

^a^Reference = avocado non-consumer.

^b^Model 1: Unadjusted.

^c^Model 2: Adjusted for maternal age at delivery, marital status, education, nulliparity, BMI in the first trimester^,^ gestational age at delivery, caesarean section, neonatal intensive care unit admission, breastfeeding, alcohol consumption, EPDS, diet quality (AHEI-P) and smoking.

## Discussion

This analysis used a prospective cohort study to investigate the link between maternal avocado consumption during pregnancy and the offspring’s allergic health outcomes. The findings indicate that 12-month-old infants have lower odds of developing food allergies when their mothers consumed avocados during pregnancy. Importantly, this association remained significant even after adjusting for relevant confounding variables including maternal age at delivery, marital status, education, nulliparity, BMI in the first trimester, gestational age at delivery, caesarean section, neonatal intensive care unit admission, breastfeeding, alcohol consumption, postpartum depression, diet quality, and smoking, which have been shown to have an impact on the development of food allergies. Similar findings were also noted with paroxysmal wheezing, but the association attenuated in the fully adjusted model. No significant associations were observed between maternal avocado consumption and the other available allergic health outcomes (rhinitis and eczema) in their offspring.

While our findings are the first to report on maternal avocado consumption and offspring allergy, decades of previous research have explored the relationship between maternal diet, in terms of specific dietary patterns, food groups, and food items, and offspring allergic outcomes.^1,18–23^ Our findings align with the previous research on vegetarian and Mediterranean dietary patterns. First, one avocado provides 13.3 grams of monounsaturated fatty acids and 9.5 grams of dietary fiber; and its nutrient profile aligns well with the Mediterranean and vegetarian diets.^24^ Moreover, studies suggest that maternal Mediterranean dietary patterns are associated with fewer allergic outcomes in offspring. For example, Chatzi et al. studied 460 mother and child pairs in Spain and found that mothers with higher Mediterranean Diet Score during pregnancy had lower odds of their offspring developing wheezing and atopy after 6.5 years of follow-up while controlling for relevant covariates.^6^ The Japan Environment and Children’s Study also observed similar results, which found that those with higher adherence to the Mediterranean diet during pregnancy had a lower risk of their children developing asthma or one or more allergies at four years old.^25^ Furthermore, the Taiwan Birth Study observed a lower incidence of child eczema before 18 months among mothers who followed a vegetarian diet during pregnancy.^26^ However, two studies did not find any association between maternal dietary patterns during pregnancy and allergic health outcomes among their offspring.^22,23^

Consuming certain food groups has also been associated with a lower risk of allergic health outcomes among offspring. In the EDEN birth cohort with 1140 mother and child pairs, higher consumption of raw vegetables (which included avocado) during pregnancy was associated with a lower risk of allergic rhinitis among offspring at three years old.^27^ Similar results with fruits and vegetables were also noted in the Finnish Type 1 Diabetes Prediction and Prevention Nutrition Study^28^ and a Japanese cohort.^29^

However, fewer studies have looked at individual food items and their impact on allergies. Willers et al. found that maternal apple intake during pregnancy was associated with lower wheezing and asthma among offspring at five years old.^8^ Another study found that children born to mothers who consumed legumes once a month or less were at a heightened risk of belonging to the ‘multi-allergic’ cluster, suggesting a potential role for legume consumption in preventing allergic diseases.^30^ A third study observed an inverse association between maternal intake of peanuts and tree nuts and the prevalence of asthma in 18-month-old toddlers.^31^ The dietary pattern and food group research is likely confounded by each food’s unique nutrient and bioactive composition. Overall, very little data exists to support specific food recommendations for maternal diets to impact allergies.

The potential benefit of avocados during pregnancy might be explained by various mechanisms at the nutrient level (e.g., antioxidants, fiber, and monounsaturated fat). First, researchers hypothesized that prenatal antioxidant exposure may program the child’s susceptibility to allergic health outcomes *in utero* through changing T-cell responses.^32,33^ Although results are mixed, some existing observational studies have shown an inverse relationship between offspring allergic health outcomes and antioxidant consumption during pregnancy, including vitamin E^2,34–36^ and zinc.^2,35^ One avocado provides 2.6 mg of vitamin E (18% of the Daily Value) and 0.93 mg of zinc.^37^ Secondly, one avocado also provides 9.25 grams of fiber,^37^ which has been shown to be a significant factor in shaping the gastrointestinal tract microbial community, microbial fermentation, and subsequent host immune maturation.^19^ Preclinical studies have shown that maternal gut microbial-produced short-chain fatty acids from fiber consumption during pregnancy modulate key immune pathways via epigenetic effects in the fetus, ultimately improving or preventing the offspring’s allergic responses.^38,39^ Fiber may even have a protective effect later in life. Among adults, higher fiber consumption appeared protective against wheezing and other respiratory symptoms.^40,41^ However, there are limited studies on this topic among mother-children pairs.^41,42^ Lastly, avocados are abundant in monounsaturated fats (13.3 grams in one fruit). Studies have shown that monounsaturated fats can modulate immunological response and were inversely related to asthma in both adolescents and adults.^43,44^ Similar to dietary fiber, there are limited studies on this topic among mother-child pairs.

Our results suggest that 12-month-old infants were less likely to develop food allergies when their mothers consumed avocado during pregnancy. However, no association between maternal avocado intake and the other three allergic health outcomes was found. One plausible explanation for this divergence could be the varying timelines for developing or manifesting these allergic diseases.^45^ For example, food allergies typically emerge between the ages of 6 and 12 months, coinciding with the introduction of solid foods.^45^ In contrast, only 50% of children with eczema exhibit symptoms in their first year of life, and 95% experience onset within the initial five years of life.^45^ Similarly, rhinitis also develops later in childhood.^45^ Given that our assessment was based on outcomes at 12 months of age, it is possible that some of these allergic diseases may not have manifested yet. Further research is warranted to reproduce this study with outcomes assessed at different age intervals.

This study included several strengths. First, this prospective cohort study allows a better understanding of the temporal relationship between exposure and health outcomes.^46^ Second, avocado intake was assessed with an FFQ, more representative of the usual intake of sporadically consumed foods such as avocado.^47^ Third, the final model accounted for several covariates to better comprehend the association between avocado consumption during pregnancy and allergic health outcomes in their children. However, several limitations should be highlighted. First, these analyses relied on self-reported dietary intake and health outcomes. This may introduce recall and measurement biases, leading to an underestimation of the true effect of the other potential confounding factors. Second, although this paper focused on avocado consumption, a single food item, it may be an indicator of broader vegetarian dietary patterns that are not fully characterized. However, we attempted to account for this by adjusting for AHEI-P in our models. Third, only pregnant women likely to deliver at Kuopio University Hospital were recruited and close to 50% of the original participants were excluded. This may limit the generalizability of the results. However, the characteristics of the participants were representative of the overall population of women who gave birth in Finland.^12^ Lastly, although we adjusted for relevant covariates, an observational study design may be susceptible to residual confounding.

## Conclusions

This prospective cohort study demonstrated that 12-month-old infants have lower odds of developing food allergies if their mothers consumed avocado during pregnancy. The association persisted even when accounting for potential covariates. Future studies are warranted to explore this association in other populations.

## Supplementary information

STROBE_checklist_v4_combined_PlosMedicine

## Data Availability

The datasets generated during and/or analysed during the current study are not publicly available due to the risk of identifying patients but are available from the corresponding author on reasonable request.

## References

[1] Venter, C. et al. Dietary factors during pregnancy and atopic outcomes in childhood: A systematic review from the European Academy of Allergy and Clinical Immunology. *Pediatr. Allergy Immunol.* **31**, 889–912 (2020). 32524677 10.1111/pai.13303 PMC9588404

[2] Beckhaus, A. A. et al. Maternal nutrition during pregnancy and risk of asthma, wheeze, and atopic diseases during childhood: a systematic review and meta-analysis. *Allergy* **70**, 1588–1604 (2015). 26296633 10.1111/all.12729

[3] Kim, Y. H. et al. Maternal perinatal dietary patterns affect food allergy development in susceptible infants. *J. Allergy Clin. Immunology: Pract.* **7**, 2337–2347.e7 (2019). 30930272 10.1016/j.jaip.2019.03.026

[4] Chen, L.-W. et al. Maternal dietary inflammatory potential and quality are associated with offspring asthma risk over 10-year follow-up: the Lifeways Cross-Generation Cohort Study. *Am. J. Clin. Nutr.* **111**, 440–447 (2020). 31826246 10.1093/ajcn/nqz297

[5] Venter, C., Eyerich, S., Sarin, T. & Klatt, K. C. Nutrition and the immune system: a complicated tango. *Nutrients* **12**, 818 (2020). 32204518 10.3390/nu12030818 PMC7146186

[6] Chatzi, L. et al. Mediterranean diet in pregnancy is protective for wheeze and atopy in childhood. *Thorax* **63**, 507–513 (2008). 18198206 10.1136/thx.2007.081745

[7] van Neerven, R. J. J. & Savelkoul, H. Nutrition and allergic diseases. *Nutrients* **9**, 762 (2017). 28714911 10.3390/nu9070762 PMC5537876

[8] Willers, S. M. et al. Maternal food consumption during pregnancy and asthma, respiratory and atopic symptoms in 5-year-old children. *Thorax* **62**, 773–779 (2007). 17389754 10.1136/thx.2006.074187 PMC2117307

[9] Dreher, M. L. & Davenport, A. J. Hass avocado composition and potential health effects. *Crit. Rev. Food Sci. Nutr.* **53**, 738–750 (2013). 23638933 10.1080/10408398.2011.556759 PMC3664913

[10] Dreher, M. L. & Ford, N. A. A comprehensive critical assessment of increased fruit and vegetable intake on weight loss in women. *Nutrients* **12**, 1919 (2020). 32610460 10.3390/nu12071919 PMC7399879

[11] Comerford, K. B., Ayoob, K. T., Murray, R. D. & Atkinson, S. A. The role of avocados in maternal diets during the periconceptional period, pregnancy, and lactation. *Nutrients* **8**, 313 (2016). 27213449 10.3390/nu8050313 PMC4882725

[12] Huuskonen, P. et al. Kuopio birth cohort - design of a Finnish joint research effort for identification of environmental and lifestyle risk factors for the wellbeing of the mother and the newborn child. *BMC Preg. Childbirth* **18**, 381 (2018). 10.1186/s12884-018-2013-9 PMC6150990 30241516

[13] Etusivu - Fineli. https://fineli.fi/fineli/fi/index.

[14] Männistö, S., Virtanen, M., Mikkonen, T. & Pietinen, P. Reproducibility and validity of a food frequency questionnaire in a case-control study on breast cancer. *J. Clin. Epidemiol.* **49**, 401–409 (1996). 8621990 10.1016/0895-4356(95)00551-x

[15] Cox, J. L., Holden, J. M. & Sagovsky, R. Detection of postnatal depression. Development of the 10-item Edinburgh Postnatal Depression Scale. *Br. J. Psychiatry* **150**, 782–786 (1987). 3651732 10.1192/bjp.150.6.782

[16] McCullough, M. L. et al. Diet quality and major chronic disease risk in men and women: moving toward improved dietary guidance. *Am. J. Clin. Nutr.* **76**, 1261–1271 (2002). 12450892 10.1093/ajcn/76.6.1261

[17] Rifas-Shiman, S. L., Rich-Edwards, J. W., Kleinman, K. P., Oken, E. & Gillman, M. W. Dietary quality during pregnancy varies by maternal characteristics in Project Viva: a US cohort. *J. Am. Diet. Assoc.* **109**, 1004–1011 (2009). 19465182 10.1016/j.jada.2009.03.001 PMC4098830

[18] Trikamjee, T., Comberiati, P., D’Auria, E., Peroni, D. & Zuccotti, G. V. Nutritional factors in the prevention of atopic dermatitis in children. Front. Pediatr. **8**, 577413 (2021). 10.3389/fped.2020.577413 PMC7874114 33585361

[19] Vassilopoulou, E., Guibas, G. V. & Papadopoulos, N. G. Mediterranean-type diets as a protective factor for asthma and atopy. *Nutrients* **14**, 1825 (2022). 35565792 10.3390/nu14091825 PMC9105881

[20] Netting, M. J., Middleton, P. F. & Makrides, M. Does maternal diet during pregnancy and lactation affect outcomes in offspring? A systematic review of food-based approaches. *Nutrition* **30**, 1225–1241 (2014). 25280403 10.1016/j.nut.2014.02.015

[21] Garcia-Larsen, V. et al. Diet during pregnancy and infancy and risk of allergic or autoimmune disease: A systematic review and meta-analysis. *PLoS Med* **15**, e1002507 (2018). 29489823 10.1371/journal.pmed.1002507 PMC5830033

[22] Lange, N. E. et al. Maternal dietary pattern during pregnancy is not associated with recurrent wheeze in children. *J. Allergy Clin. Immunol.* **126**, 250–255, 255.e1–4 (2010). 10.1016/j.jaci.2010.05.009 PMC2917539 20584543

[23] Sivula, E., Puharinen, H., Hantunen, S., Keski-Nisula, L. & Backman, K. Maternal dietary indexes are not linked to early childhood wheezing or atopic eczema. *Pediatr. Allergy Immunol.* **35**, e14099 (2024). 38425169 10.1111/pai.14099

[24] Ford, N. A. & Liu, A. G. The forgotten fruit: A case for consuming avocado within the traditional Mediterranean diet. *Front Nutr.* **7**, 78 (2020). 32548125 10.3389/fnut.2020.00078 PMC7272688

[25] Nakano, K. et al. Relationship between the Mediterranean diet score in pregnancy and the incidence of asthma at 4 years of age: The Japan Environment and Children’s Study. *Nutrients* **15**, 1772 (2023). 37049612 10.3390/nu15071772 PMC10096633

[26] Su, Y.-C., Xie, J.-S., Jan, R.-H. & Hsieh, C.-J. Association between a maternal vegetarian diet during pregnancy and the occurrence of atopic dermatitis in children. *Pediatr. Allergy Immunol.* **34**, e14052 (2023). 38146115 10.1111/pai.14052

[27] Baïz, N. et al. Maternal diet before and during pregnancy and risk of asthma and allergic rhinitis in children. *Allergy Asthma Clin. Immunol.* **15**, 40 (2019). 31285746 10.1186/s13223-019-0353-2 PMC6589169

[28] Erkkola, M. et al. Risk of asthma and allergic outcomes in the offspring in relation to maternal food consumption during pregnancy: a Finnish birth cohort study. *Pediatr. Allergy Immunol.* **23**, 186–194 (2012). 22432883 10.1111/j.1399-3038.2012.01272.x

[29] Miyake, Y., Sasaki, S., Tanaka, K. & Hirota, Y. Consumption of vegetables, fruit, and antioxidants during pregnancy and wheeze and eczema in infants. *Allergy* **65**, 758–765 (2010). 20102358 10.1111/j.1398-9995.2009.02267.x

[30] Delvert, R. et al. Maternal diet quality during pregnancy and allergic and respiratory multimorbidity clusters in children from the EDEN mother–child cohort. *Nutrients* **15**, 146 (2022). 36615802 10.3390/nu15010146 PMC9824220

[31] Maslova, E. et al. Peanut and tree nut consumption during pregnancy and allergic disease in children—should mothers decrease their intake? Longitudinal evidence from the Danish National Birth Cohort. *J. Allergy Clin. Immunol.* **130**, 724–732 (2012). 22743306 10.1016/j.jaci.2012.05.014

[32] Prescott, S. L. et al. Development of allergen-specific T-cell memory in atopic and normal children. *Lancet* **353**, 196–200 (1999). 9923875 10.1016/S0140-6736(98)05104-6

[33] Devereux, G. Early life events in asthma-diet. *Pediatr. Pulmonol.* **42**, 663–673 (2007). 17595038 10.1002/ppul.20640

[34] Martindale, S. et al. Antioxidant intake in pregnancy in relation to wheeze and eczema in the first two years of life. *Am. J. Respir. Crit. Care Med.* **171**, 121–128 (2005). 15531754 10.1164/rccm.200402-220OC

[35] Litonjua, A. A. et al. Maternal antioxidant intake in pregnancy and wheezing illnesses in children at 2 y of age. *Am. J. Clin. Nutr.* **84**, 903–911 (2006). 17023719 10.1093/ajcn/84.4.903 PMC1994925

[36] Devereux, G. et al. Low maternal vitamin E intake during pregnancy is associated with asthma in 5-year-old children. *Am. J. Respir. Crit. Care Med.* **174**, 499–507 (2006). 16763215 10.1164/rccm.200512-1946OC

[37] FoodData Central. https://fdc.nal.usda.gov/fdc-app.html#/food-details/171706/nutrients.

[38] Thorburn, A. N. et al. Evidence that asthma is a developmental origin disease influenced by maternal diet and bacterial metabolites. *Nat. Commun.* **6**, 7320 (2015). 26102221 10.1038/ncomms8320

[39] Hogenkamp, A., Thijssen, S., van Vlies, N. & Garssen, J. Supplementing pregnant mice with a specific mixture of nondigestible oligosaccharides reduces symptoms of allergic asthma in male offspring. *J. Nutr.* **145**, 640–646 (2015). 25733483 10.3945/jn.114.197707

[40] Saeed, M. A. et al. Association of dietary fiber on asthma, respiratory symptoms, and inflammation in the adult National Health and Nutrition Examination Survey population. *Ann. Am. Thorac. Soc.* **17**, 1062–1068 (2020). 32369709 10.1513/AnnalsATS.201910-776OC

[41] Sdona, E., Georgakou, A. V., Ekström, S. & Bergström, A. Dietary fibre intake in relation to asthma, rhinitis and lung function impairment—A systematic review of observational studies. *Nutrients* **13**, 3594 (2021). 34684594 10.3390/nu13103594 PMC8539618

[42] Pretorius, R. A., Bodinier, M., Prescott, S. L. & Palmer, D. J. Maternal fiber dietary intakes during pregnancy and infant allergic disease. *Nutrients* **11**, 1767 (2019). 31374861 10.3390/nu11081767 PMC6722741

[43] Troisi, R. J. et al. A prospective study of diet and adult-onset asthma. *Am. J. Respir. Crit. Care Med* **151**, 1401–1408 (1995). 7735592 10.1164/ajrccm.151.5.7735592

[44] Huang, S. L. & Pan, W. H. Dietary fats and asthma in teenagers: analyses of the first Nutrition and Health Survey in Taiwan (NAHSIT). *Clin. Exp. Allergy* **31**, 1875–1880 (2001). 11737039 10.1046/j.1365-2222.2001.01222.x

[45] Thomsen, S. F. Epidemiology and natural history of atopic diseases. *Eur. Clin. Respir. J* **2**, 10.3402/ecrj.v2.24642 (2015). 10.3402/ecrj.v2.24642 PMC4629767 26557262

[46] Carlson, M. D. A. & Morrison, R. S. Study design, precision, and validity in observational studies. *J. Palliat. Med.* **12**, 77–82 (2009). 19284267 10.1089/jpm.2008.9690 PMC2920077

[47] Shim, J.-S., Oh, K. & Kim, H. C. Dietary assessment methods in epidemiologic studies. *Epidemiol. Health* **36**, e2014009 (2014). 25078382 10.4178/epih/e2014009 PMC4154347

